# Elucidating Immune Cell Mediated Causal Pathways Linking Blood Metabolites to Major Depressive Disorder: A Mediation Mendelian Randomization Analysis

**DOI:** 10.1002/brb3.71421

**Published:** 2026-06-08

**Authors:** Zhiwei Xu, Yining Zhou, Xuecheng Zhang, Xiaowen Chen, Yajing Li, Jintao Wang, Ting Chang, Zhen Yuan, Hongling Jia

**Affiliations:** ^1^ Shandong University of Traditional Chinese Medicine Jinan Shandong China; ^2^ Second Affiliated Hospital of Shandong University of Traditional Chinese Medicine Jinan Shandong China; ^3^ China–Japan Friendship Hospital Beijing China; ^4^ Beijing Tsinghua Changgung Hospital Beijing China; ^5^ Shanghai University of Traditional Chinese Medicine Shanghai China

**Keywords:** immune cells, major depressive disorder, mediation, metabolites

## Abstract

**Background:**

Metabolic and immune alterations have been reported in patients with major depressive disorder (MDD), yet the biological pathways linking these changes to MDD remain incompletely understood. This study aims to examine the association between blood metabolites and the risk of MDD, investigate the mediating role of immune cells, and explore their causal relationships.

**Methods:**

We analyzed genome‐wide association study (GWAS) summary statistics for 1400 circulating metabolites and 731 immune‐cell traits, together with an MDD GWAS comprising 135,458 cases and 344,901 controls, all obtained from the GWAS Catalog and the Psychiatric Genomics Consortium. Causal effects of each metabolite on MDD were estimated with seven Mendelian randomization (MR) approaches: inverse variance weighted, MR‐Egger, weighted median, debiased inverse variance weighted method (IVW), Bayesian weighted MR, Mendelian randomization robust adjusted profile score (MR‐RAPS), and constrained maximum likelihood and model averaging. Horizontal pleiotropy was evaluated with Mendelian Randomization Pleiotropy RESidual Sum and Outlier method (MR‐PRESSO), RadialMR, and the MR‐Egger intercept, whereas Cochran's *Q* statistic and leave‐one‐out analyses assessed heterogeneity and sensitivity. Finally, MR‐based mediation analysis quantified the extent to which immune‐cell traits mediate the metabolite–MDD associations.

**Results:**

MR analyses provided evidence consistent with potential causal effects of 10 metabolites, including 1‐(1‐enyl‐stearoyl)‐2‐linoleoyl‐GPE, 2‐linoleoylglycerol, kynurenine, spermidine‐to‐histidine ratio, 1‐linoleoylglycerol, 4‐vinylguaiacol sulfate, dopamine 4‐sulfate, *N*‐acetyl‐aspartyl‐glutamate, sphingomyelin and taurine to glutamate ratio and MDD. Mediation analyses identified three immune‐cell‐mediated pathways: CD8dim T cells mediated 5% of the effect of 2‐linoleoylglycerol on MDD risk; CD11b expression on basophils mediated 19% of the effect of dopamine 4‐sulfate on MDD risk; and CD4 expression on resting regulatory CD4 T cells mediated 14% of the effect of the spermidine‐to‐histidine ratio on MDD risk.

**Conclusion:**

Our findings suggest that specific metabolites may affect the risk of MDD via immune‐related pathways.

## Graphical abstract

Our integrative Mendelian randomization framework revealed that specific circulating metabolites exert causal effects on major depressive disorder, partly through immune‐cell–mediated pathways. These findings highlight metabolite–immune interactions as potential mechanistic targets for MDD prevention and therapy.

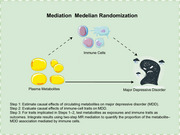

**Abbreviations**
BWMR: Bayesian weighted Mendelian randomization
cML‐MA: constrained maximum likelihood and model averaging
CNS: central nervous system
dIVW: debiased inverse variance weighted method
IVs: instrumental variables
IVW: inverse variance weighted method
MDD: major depressive disorder
MR: Mendelian randomization
MR‐Egger: Mendelian randomization Egger regression
MR‐PRESSO: Mendelian randomization Pleiotropy RESidual Sum and Outlier method
MR‐RAPS: Mendelian randomization robust adjusted profile score
mtDNA: mitochondrial DNA
SNPs: single nucleotide polymorphisms
STROBE: strengthening the reporting of observational studies in epidemiology
Weighted Median: weighted median estimator

## 1 Introduction

Major depressive disorder (MDD) is a prevalent psychiatric condition affecting approximately 300 million individuals worldwide, with a global prevalence of approximately 4.7% (Marx et al. 2023; Ferrari et al. 2013; Otte et al. 2016). MDD is a leading cause of disability worldwide and is expected to remain a major contributor to the global burden of disease (Malhi and Mann 2018). The disorder is associated with significant disability and a high rate of suicidal behavior, reaching up to 53.1% among patients (Dong et al. 2018), highlighting its status as a critical global public health concern. Moreover, up to one‐third of patients exhibit resistance to conventional antidepressants (Rush et al. 2006). These challenges underscore the necessity for a deeper understanding of the underlying mechanisms of MDD and the identification of potential biological markers.

Increasing evidence suggests that metabolic dysregulation is widespread in MDD. Among these alterations, lipid metabolism is notably affected, with large‐scale metabolomic studies demonstrating downregulation of polyunsaturated fatty acids, long‐chain monounsaturated fatty acids, and long‐chain saturated fatty acids in patients with MDD (Pu et al. 2021; Jansen et al. 2024). Additionally, amino acid metabolism disruptions are prevalent; comprehensive evaluations of the plasma neurotransmitter metabolomic profile in MDD have revealed dysregulation of γ‐aminobutyric acid, dopamine, tyramine, and kynurenine levels (Pan et al. 2018). These metabolites may serve as plasma biomarkers to distinguish individuals with MDD from healthy controls. Furthermore, a Mendelian randomization (MR) study identified 34 metabolites, including lipids and amino acids, that have causal relationships with MDD (Dong et al. 2024).

In parallel, MDD is characterized by activated bidirectional signaling between the central nervous system (CNS) and the immune system (Weber et al. 2017). This is evidenced by increased activation of central microglia and alterations in the proportions of certain peripheral immune cell types (Drevets et al. 2022). Notably, patients with MDD exhibit significant changes in T cell populations, including reduced proportions of CD4 T cells and elevated levels of regulatory T cells and CD8 T cells (Sun et al. 2024; Suzuki et al. 2017). These alterations suggest that dysregulated T cell activation may influence neuroinflammatory processes and neuroplasticity‐related pathways in MDD (Toben and Baune 2015). Supporting this notion, a study by Ronaldson et al. showed that an elevated percentage of Treg cells was associated with greater depressive symptom severity (Ronaldson et al. 2016). Moreover, an MR study provided preliminary evidence that 29 immune phenotypes, including CD4 and CD8 T cells, are causally linked to the risk of MDD (Zhang et al. 2024).

Emerging evidence indicates that metabolic reprogramming is critical for immune‐cell differentiation and function. Notably, lipid mediators have been shown to directly influence T cell function. For instance, short‐chain fatty acids produced by the gut microbiota play a crucial role in the induction and homeostasis of regulatory T cells (Arpaia et al. 2013; Furusawa et al. 2013; Smith et al. 2013). Similarly, amino acids are vital for T cell activation, differentiation, and proliferation (Hope and Salmond 2021). Enzymes responsible for the breakdown and metabolism of amino acids are critical in regulating T cell activation and signaling by modifying key pathways such as the T cell receptor and co‐stimulation (Castellano and Molinier‐Frenkel 2020). Although current research has identified associations between peripheral metabolites and immune cells in patients with MDD, the intricate relationships of immune metabolism within the context of MDD remain largely unexplored. Furthermore, it is still unclear whether immune‐cell traits mediate the effects of metabolites on MDD risk.

MR employs germline genetic variants as instrumental variables (IVs) to infer causal relationships while minimizing confounding (Smith and Ebrahim 2003). In this study, we used MR to investigate the potential causal effects of circulating metabolites on MDD risk and to determine whether immune‐cell traits mediate any identified effects.

## 2 Methods

### 2.1 Study Design

**FIGURE 1:**
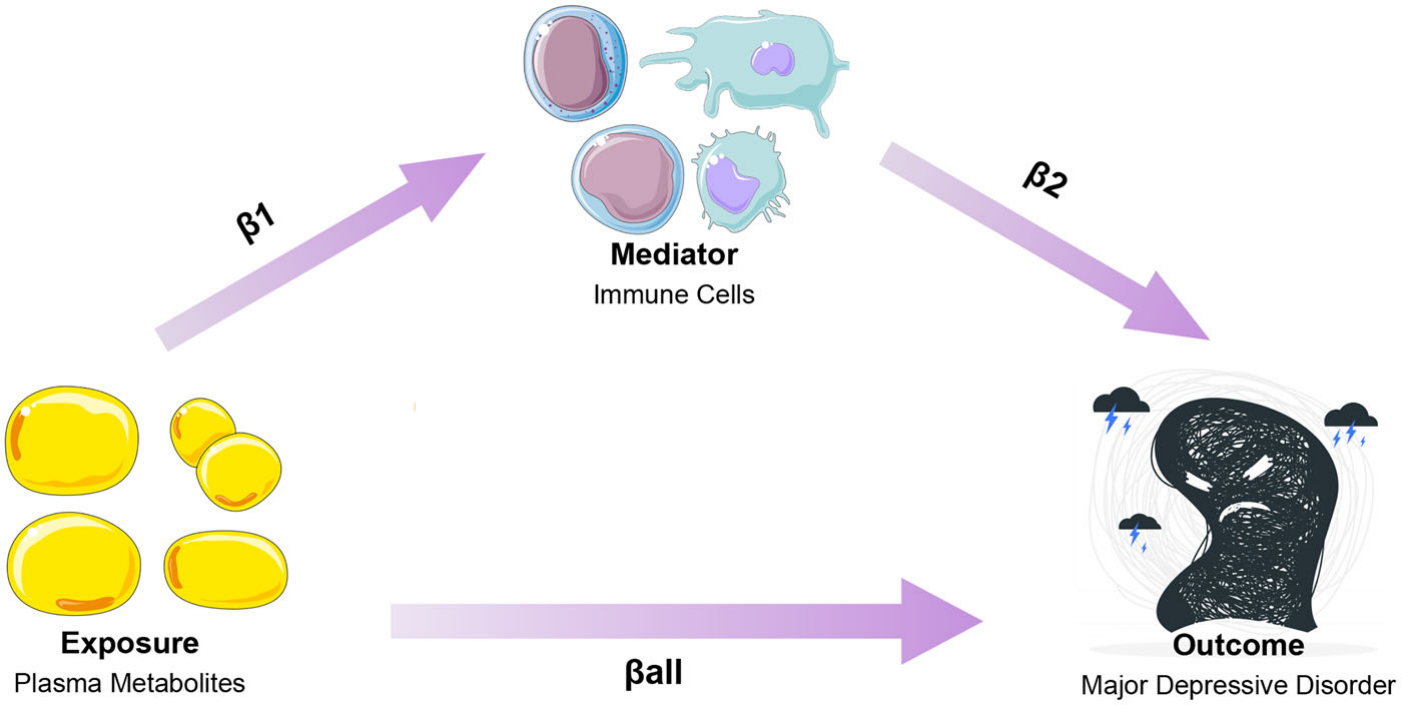
Study design flowchart.

The MR analysis proceeded in three stages. We first evaluated the total effect of each circulating metabolite on MDD. We then tested the causal effects of immune‐cell traits on MDD. For metabolites associated with MDD and immune‐cell traits associated with MDD, we next estimated the effect of each metabolite on the corresponding immune‐cell trait. Metabolite–immune–MDD triplets meeting these criteria were subsequently carried forward into the two‐step MR mediation analysis (Figure 1).

### 2.2 Genome‐Wide Association Study (GWAS) Sources

MDD data were sourced from the Psychiatric Genomics Consortium (https://pgc.unc.edu/), the largest biological investigation in psychiatry. The consortium performed a genome‐wide association meta‐analysis using seven cohorts, which included 135,458 cases and 344,901 controls, and analyzed 9.6 million single nucleotide polymorphisms (SNPs) (Wray et al. 2018). Metabolite data were obtained from a GWAS involving 8299 unrelated European participants (Chen et al. 2023), examining 1091 blood metabolites and 309 metabolite ratios. Among these, 850 plasma metabolites were classified into eight categories: lipid, amino acid, xenobiotics, nucleotide, cofactors and vitamins, carbohydrate, peptide, and energy. The remaining 241 metabolites were designated as unknown or partially characterized molecules. Immune cell data were acquired from a GWAS of a general population cohort consisting of 3757 individuals from Sardinia (Orrù et al. 2020). The study assessed 731 immune traits, including 118 absolute cell counts, 389 surface antigen mean fluorescence intensities, 32 morphological parameters, and 192 relative counts. All subjects in these studies were of European ancestry (Table 1). We assessed the likelihood of sample overlap across the exposure, mediator, and outcome GWAS datasets. On the basis of the original study descriptions, no substantial sample overlap was evident/sample overlap could not be completely excluded.

**TABLE 1:** Genome‐wide association study (GWAS) data sources.

Phenotype	Sample size	Race	PMID
Metabolites	8299	European	PMC7614162
Immune cells	3757	European	PMC8517961
MDD	135458MDD and 344901HC	European	PMC5934326

Abbreviations: HC, healthy control; MDD, major depressive disorder.

### 2.3 IVs Selection

MR relies on three core assumptions (Richmond and Davey Smith 2022): relevance (Marx et al. 2023), meaning that the genetic instruments are robustly associated with the exposure; independence (Ferrari et al. 2013), meaning that the instruments are not associated with confounders of the exposure–outcome relationship; and exclusion restriction (Otte et al. 2016), meaning that the instruments affect the outcome only through the exposure. For each exposure, we selected SNPs strongly associated with the exposure as IVs, using a significance threshold of *p* < 5 × 10^−6^. To account for linkage disequilibrium, we excluded SNPs within a 10,000 kb window that had an *r* ^2^ > 0.001 with the most significant SNP, ensuring that the selected IVs were independent. To assess and mitigate weak instrument bias, we calculated the *F*‐statistic (*F* = (*β*/SE)^2^) for each SNP (Huang et al. 2021). By applying an *F*‐statistic filter, we reduced the influence of weak instruments on the results. SNPs meeting all the above criteria were further assessed for their associations with outcome risk factors to minimize the impact of pleiotropy.

### 2.4 Statistical Analysis

We employed multiple methods for MR analysis to confirm the reliability of our results. The primary method was the random effect inverse variance weighted (IVW) method, which presumes all IVs are valid and combines estimates by weighting them with the inverse of their variance, thus offering substantial statistical power (Hemani et al. 2018). We also applied the following methods. The MR‐Egger approach permits directional pleiotropy by estimating an intercept term, which can signal the existence of pleiotropy (Bowden et al. 2015). The weighted median method yields consistent estimates even when up to half of the IVs are invalid by determining the median of the IV estimates weighted by their inverse variance (Bowden et al. 2016). We also employed the debiased IVW (dIVW) method, an adaptation of the IVW approach that incorporates a bias correction factor to improve resilience against weak instruments (Ye et al. 2021). Additionally, the Bayesian weighted MR (BWMR) is a Bayesian approach that addresses violations of IV assumptions due to pleiotropy, allowing for causal inference even in its presence (Zhao, Ming, et al. 2020). The Mendelian randomization robust adjusted profile score (MR‐RAPS) method directly addresses both systematic and idiosyncratic pleiotropic effects, ensuring accurate MR analyses even when numerous weak instruments are present (Zhao, Wang, et al. 2020). The constrained maximum likelihood and model averaging (cML‐MA) method efficiently detects invalid IVs, providing trustworthy causal effect estimates regardless of the influence of pleiotropic effects (Xue et al. 2021).

To evaluate and mitigate potential pleiotropy, we applied the following methods. Initially, the MR Pleiotropy RESidual Sum and Outlier (MR‐PRESSO) method was used to identify and correct outlier IVs indicative of horizontal pleiotropy (Verbanck et al. 2018). Any detected outliers were removed from the analysis. Additionally, we applied the RadialMR method to further identify and eliminate outliers (Bowden et al. 2018). Within the MR‐Egger framework, the intercept term can reveal the presence of horizontal pleiotropy; a significant MR‐Egger intercept test (*p* < 0.05) indicates pleiotropy. We also employed Cochran's *Q* test to evaluate heterogeneity. Sensitivity analyses were conducted using the leave‐one‐out approach and funnel plots to assess the stability of the findings.

We then applied a two‐step MR mediation framework. We denoted the causal effect of each metabolite on the immune‐cell trait as *β*_1_, the effect of the immune‐cell trait on MDD as *β*_2_, and the total effect of the metabolite on MDD as *β*_total. The indirect effect mediated by immune cells was estimated as *β*_indirect = *β*_1_ × *β*_2_, and the mediation proportion as *β*_indirect/*β*_total. Standard errors and 95% confidence intervals for *β*_indirect and the mediation proportion were obtained with the Delta method, and *p* values were derived under a normality assumption.

The IVW estimate was treated as the primary result, and FDR correction was applied to IVW‐derived *p* values to account for multiple testing. Findings were considered robust when the IVW result passed FDR correction, the effect directions were consistent across sensitivity analyses, and there was no strong evidence of horizontal pleiotropy or substantial heterogeneity.

### 2.5 Software

All analyses were conducted using R software (version 4.3.1), utilizing packages such as TwoSampleMR, mediation, ggplot2, and forestploter.

## 3 Results

### 3.1 MR Study

#### 3.1.1 Associations Between Metabolites and MDD

**FIGURE 2:**
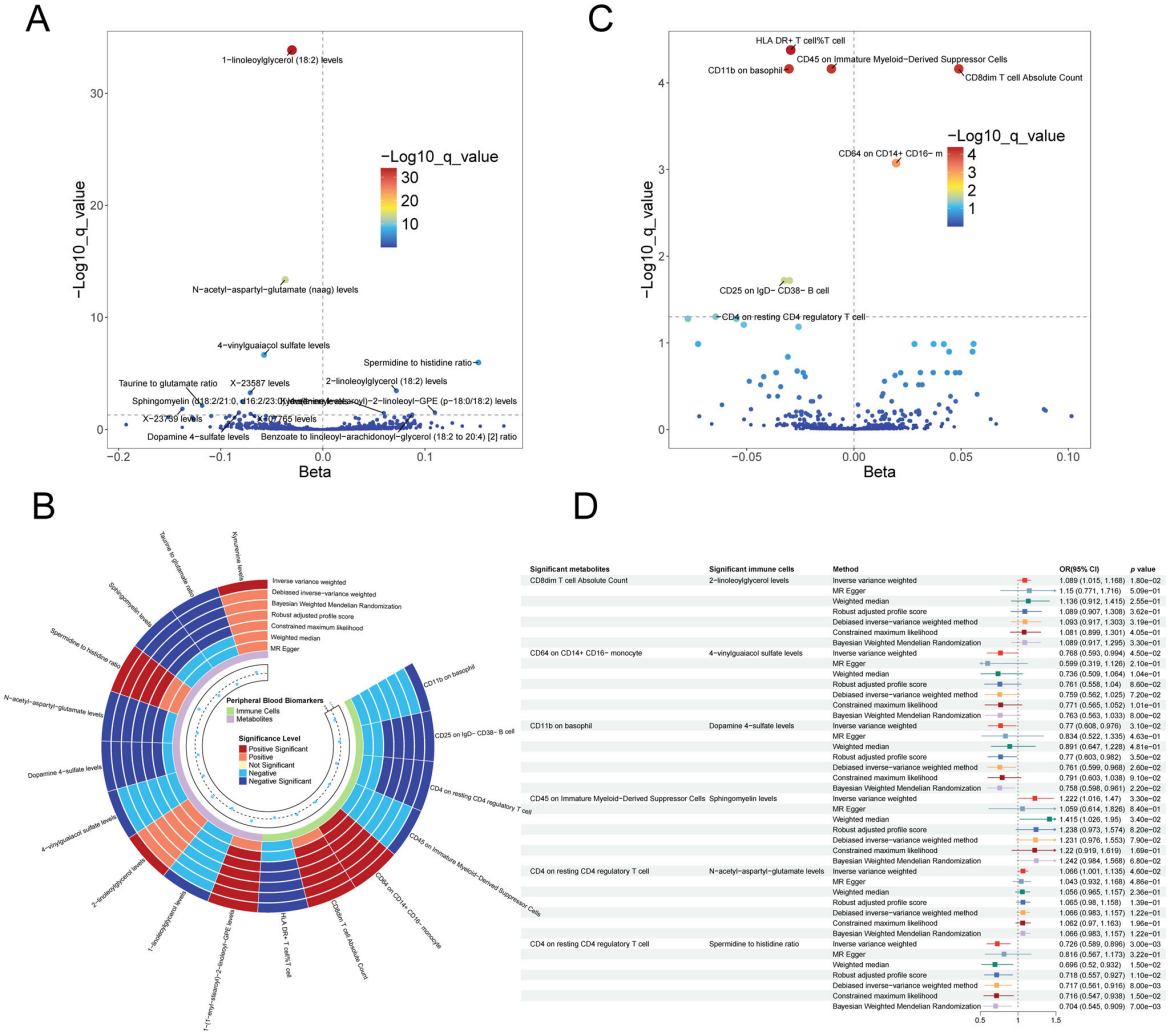
MR results: (A) IVW results for associations of metabolites to major depressive disorder (MDD); (B) IVW results for associations of immune cells to MDD; (C) MR analysis of immune cells to metabolites in relation to MDD; and (D) associations of metabolites to immune cells. MR, Mendelian randomization.

We manually searched the GWAS Catalog website and excluded 86 confounding SNPs (Table S1). The final IVs are presented in Table S2, with all *F*‐statistics above 10. After FDR correction, 14 metabolites were significantly associated with MDD (Figure 2A). In the reverse analysis, a causal relationship between MDD and benzoate to linoleoyl‐arachidonoyl‐glycerol ratio was identified (*β* = −0.145, 95% CI: −0.247 to −0.0436, *p* = 0.005), resulting in the exclusion of it from subsequent analyses (Table S3). X‐07765, X‐23587, and X‐23739 levels are categorized as an unknown metabolite and were similarly excluded from subsequent analyses.

Of the remaining 10 significant metabolites, 4 types increased the risk of MDD, including 1‐(1‐enyl‐stearoyl)‐2‐linoleoyl‐GPE levels (OR = 1.116, 95% CI: 1.052–1.183, P.FDR = 0.031), 2‐linoleoylglycerol levels (OR = 1.075, 95% CI: 1.044–1.106, P.FDR < 0.001), kynurenine levels (OR = 1.061, 95% CI: 1.028–1.096, P.FDR = 0.034), and spermidine‐to‐histidine ratio (OR = 1.164, 95% CI: 1.107–1.224, P.FDR < 0.001). However, six types decreased the risk of MDD, including 1‐linoleoylglycerol levels (OR = 0.97, 95% CI: 0.966–0.975, P.FDR < 0.001), 4‐vinylguaiacol sulfate levels (OR = 0.944, 95% CI: 0.927–0.961, P.FDR < 0.001), dopamine 4‐sulfate levels (OR = 0.921, 95% CI: 0.882–0.962, P.FDR = 0.031), *N*‐acetyl‐aspartyl‐glutamate levels (OR = 0.964, 95% CI: 0.955–0.972, P.FDR < 0.001), sphingomyelin levels (OR = 0.924, 95% CI: 0.892–0.958, P.FDR = 0.003), and taurine‐to‐glutamate ratio (OR = 0.888, 95% CI: 0.84–0.94, P.FDR = 0.007).

Sensitivity analysis revealed that the estimated directions were consistent across multiple statistical methods (Figure 2B), with no significant heterogeneity or pleiotropy detected (Tables S4 and S5, Figures S1 and S2).

#### 3.1.2 Associations Between Immune Cells and MDD

After manually excluding 37 confounding SNPs through a search of the GWAS Catalog website (Table S1), the final IVs are presented in Table S2, with all *F*‐statistics above 10. After applying FDR correction, seven immune cells showed significant associations with MDD (Figure 2C). Reverse‐direction MR did not identify associations meeting the prespecified threshold (Table S3).

Of the seven immune cells, two types were found to increase the risk of MDD, including CD64 on CD14+ CD16− monocyte (OR = 1.02, 95% CI: 1.011–1.029, P.FDR < 0.001) and CD8dim T cell absolute count (OR = 1.05, 95% CI: 1.031–1.07, P.FDR < 0.001). However, five types were found to decrease the risk of MDD, including CD11b on basophil (OR = 0.97, 95% CI: 0.959–0.982, P.FDR < 0.001), CD25 on IgD–CD38–B cell (OR = 0.968, 95% CI: 0.952–0.985, P.FDR = 0.019), CD4 on resting CD4 regulatory T cell (OR = 0.938, 95% CI: 0.904–0.972, P.FDR = 0.049), CD45 on immature myeloid‐derived suppressor cells (OR = 0.99, 95% CI: 0.986–0.994, P.FDR < 0.001), and HLA DR+ T cell% T cell (OR = 0.971, 95% CI: 0.961–0.981, P.FDR < 0.001).

Sensitivity analyses demonstrated that the direction of the estimates was consistent across multiple statistical methods (Figure 2B), and no significant heterogeneity or pleiotropy was observed (Tables S4 and S5, Figures S3 and S4).

### 3.2 Mediation MR

We then evaluated the metabolite–immune trait pairs carried forward from the previous analyses and identified six associations that met the prespecified significance criterion (Figure 2D). Reverse causation was observed between CD64 on CD14+ CD16− monocyte and 4‐vinylguaiacol sulfate levels (*β* = −0.016, 95% CI: −0.031 to −0.001, *p* = 0.042), and between CD45 on immature myeloid‐derived suppressor cells and sphingomyelin levels (*β* = −0.035, 95% CI: −0.044 to −0.026, *p* < 0.001), leading to their exclusion from subsequent analyses. For the remaining pairs, effect directions were consistent across sensitivity analyses, and there was no strong evidence of horizontal pleiotropy or substantial heterogeneity (Figure 2D, Tables S6 and S7, Figures S5 and S6).

Mediation analyses were then performed for the four retained metabolite–immune trait pairs. Among the four retained pairs, three showed evidence of significant mediation. CD8dim T cell absolute count mediated 5% of the effect of 2‐linoleoylglycerol on MDD risk; CD11b expression on basophils mediated 19% of the effect of dopamine 4‐sulfate on MDD risk; and CD4 expression on resting regulatory CD4 T cells mediated 14% of the effect of the spermidine‐to‐histidine ratio on MDD risk (Table 2).

**TABLE 2:** Mediation Mendelian randomization (MR) results.

Exposure	Mediator	Mediated effect	Mediated ratio	Mediated *p* value
2‐linoleoylglycerol levels	CD8dim T cell absolute count	0.00416	0.0578	0.03177
Dopamine 4‐sulfate levels	CD11b on basophil	0.007912	−0.0962	0.04650
*N*‐acetyl‐aspartyl‐glutamate levels	CD4 on resting CD4 regulatory T cell	−0.0041	0.19181	0.08389
Spermidine‐to‐histidine ratio	CD4 on resting CD4 regulatory T cell	0.020625	0.13550	0.02372

## 4 Discussion

In this study, we applied a two‐step MR framework to investigate potential causal associations between circulating metabolites, immune cell subsets, and MDD. Univariate analyses supported potential causal associations between ten metabolites—including 2‐linoleoylglycerol—and MDD, as well as between seven immune cell subsets—including CD8dim T cells—and MDD. Subsequent mediation analyses suggested that specific immune‐cell traits may partially mediate the associations between certain metabolites and MDD risk. Specifically, CD8dim T cells mediated 5% of the effect of 2‐linoleoylglycerol on MDD. Similarly, CD11b basophils mediated 19% of the effect of dopamine 4‐sulfate on MDD, and CD4 on resting CD4 regulatory T cells mediated 14% of the effect of the spermidine‐to‐histidine ratio on MDD. These findings enhance our understanding of the immune‐metabolic mechanisms underlying MDD and highlight potential targets for therapeutic intervention.

2‐Linoleoylglycerol acts as an antagonist of arachidonic acid glycerol ester in neurotransmission, potentially disrupting normal neurotransmission processes and thereby contributing to the symptoms of MDD (Murataeva et al. 2016). Additionally, 2‐linoleoylglycerol functions as a partial agonist of CB1 receptors within the endogenous cannabinoid system, which is crucial for emotion regulation and stress responses (Lu et al. 2019). This suggests that 2‐Linoleoylglycerol may influence emotional states, linking it to depressive symptoms. By modulating the endogenous cannabinoid system, 2‐linoleoylglycerol affects the release of inflammatory cytokines (Almogi‐Hazan and Or 2020). Given that depression is frequently associated with inflammatory responses (Miller and Raison 2016), the anti‐inflammatory and immunomodulatory properties of cannabinoids are particularly relevant. Specifically, cannabinoids inhibit T cell activation through CB2 and other receptors (Robinson et al. 2013, 2015; Börner et al. 2007), which helps explain the mediating role of CD8dim T cells. CD8dim T cells display pro‐inflammatory characteristics in certain disease states (Falanga et al. 2017; Margoles et al. 2016), such as chronic infections or immune dysregulation, and can secrete pro‐inflammatory cytokines like IFN‐γ and TNF‐α. Peripheral inflammatory signals may influence the brain through humoral, neural, and cellular pathways. Furthermore, CD8dim T cells exhibit reduced mitochondrial activity (Clutton et al. 2022). Mitochondria are essential for regulating cognitive and emotional states, and mitochondrial dysfunction has been observed in patients with MDD (Ciubuc‐Batcu et al. 2024). Such dysfunction not only impairs energy metabolism but also enhances immune inflammatory responses. Mitochondrial DNA (mtDNA), released during stress‐induced mitochondrial dysfunction, acts as an inflammatory activator and may serve as a biomarker for neuroinflammation when present in peripheral blood (Ye et al. 2025). Thus, 2‐linoleoylglycerol plays a critical role in the immune‐metabolic mechanisms of MDD by modulating CD8dim T cells and their associated mitochondrial functions.

Dopamine 4‐sulfate, a peripheral blood marker of dopamine metabolism, may reflect interactions between the CNS and the peripheral nervous system. Observational studies have found that patients with MDD exhibit impaired dopamine neurotransmission (Hasler et al. 2008), characterized by exacerbated depressive symptoms and anhedonia following catecholamine depletion. This suggests that dopamine plays a crucial role in emotion regulation and, therefore, may be indirectly relevant to dopaminergic pathways implicated in MDD. Additionally, variations in the DRD2 gene have been linked to the risk of developing MDD (Zhang et al. 2023), further supporting the connection between dopamine metabolism and neurotransmission in MDD. Dopamine receptors are expressed in nearly all immune cells (Franco et al. 2021), and dopamine can regulate processes such as antigen presentation, T cell activation, and inflammation, corroborating our findings on immune cell‐mediated mechanisms. Available evidence suggests that basophil‐related alterations are present in depression, although current findings remain limited and heterogeneous (Foley et al. 2023). Clinically, anxious depression has been associated with a reduced basophil subfraction, whereas a more recent immunophenotyping study found higher basophil counts in patients with major depressive episodes; basophil percentages also decreased after 12 weeks of SSRI treatment in adolescents with MDD, suggesting that basophil‐related signals may be treatment‐sensitive (Baek et al. 2016; Lengvenyte et al. 2025; Puangsri et al. 2023). IL‐33 directly activates human basophils via ST2, increases CD11b expression and adhesiveness, enhances migration toward eotaxin, and upregulates IL‐4 and IL‐13 expression (Suzukawa et al. 2008). These findings indicate that CD11b is an activation‐related marker on basophils and support a role for activated basophils in immune regulation (Suzukawa et al. 2008; Borriello et al. 2015). Accordingly, the inverse association between genetically predicted CD11b on basophils and MDD risk in our study may reflect a protective or compensatory basophil‐related immune phenotype. These findings suggest a potential mechanism by which dopamine metabolites influence MDD through immune regulation, thereby extending current understanding of immune‐metabolic interactions in depression. Specifically, dopamine 4‐sulfate may partly affect MDD susceptibility through a basophil‐related immune pathway.

Observational studies show that patients with MDD have significantly reduced levels of spermidine, putrescine, and serum histidine (Yazici et al. 2024; Ong et al. 2021), indicating their importance in MDD. The LHPP gene, involved in histidine dephosphorylation, is upregulated in MDD and may affect neurotransmission and synaptic plasticity by altering histidine phosphorylation, thus contributing to MDD pathogenesis (Zhuang et al. 2024; Lin et al. 2023). Spermidine promotes cellular autophagy, maintaining homeostasis (Eisenberg et al. 2009). Impaired autophagy may lead to neuronal abnormalities in MDD. Studies on natural autophagy regulators suggest that higher spermidine levels enhance autophagy (Zhou et al. 2024), improve neuronal survival and function, and reduce depression risk. Additionally, spermidine's antioxidant properties inhibit oxidative stress responses (Eisenberg et al. 2009), closely linked to MDD. Autophagy and oxidative stress jointly maintain immune cell homeostasis. Autophagy is vital for T cell development and metabolic adaptation in peripheral tissues (Bronietzki et al. 2015). In regulatory T cells, autophagy enhances mitochondrial and endoplasmic reticulum functions, ensuring self‐renewal and a resting state. Conversely, oxidative stress downregulates CD4 expression and function (Nakamura et al. 1996), disrupting T cell signaling and immune function. These findings suggest that oxidative stress and impaired autophagy may promote autoimmune and inflammatory responses by inducing cell death and functional loss, explaining our mediation results. CD4^+^ regulatory T (Treg) cells are crucial for immune tolerance. Although Treg cell numbers may increase in MDD patients (Rachayon et al. 2024; Savitz et al. 2013), their impaired function leads to immune imbalance, enhancing inflammatory responses and exacerbating depressive symptoms. In animal models of MDD, increased interleukin‐6 (IL‐6) and decreased transforming growth factor‐beta (TGF‐β) levels have been observed (Huang et al. 2022). IL‐6 induces neuroinflammation and neuronal dysfunction by disrupting the Th17/Treg balance. Dysfunctional Treg cells cannot effectively regulate this inflammation, potentially causing long‐term neuroimmune toxicity and worsening MDD. These findings suggest that the spermidine‐to‐histidine ratio may influence MDD‐related immune pathways through mechanisms related to autophagy, oxidative stress, and Treg dysfunction.

Our mediation analysis in MDD provides genetic evidence supporting the interplay between immune cells and metabolites, identifying potential biomarkers derived from immune metabolism. Unlike previous studies that have not established a direct link between immune cell metabolites and MDD, our research addresses this gap. Our findings underscore the potential relevance of immunometabolic pathways to the biological heterogeneity of MDD and may inform future mechanistic and translational research.

The study also has limitations. First, the participant group was limited to individuals of European descent, which may restrict the generalizability of our results to other ethnic populations. Future research should include diverse populations to enhance the applicability of the findings. Second, although we examined a wide range of blood metabolites, some unidentified metabolites were excluded, and the roles and mechanisms of many metabolites in MDD remain not fully understood. Lastly, despite manually excluding confounding factors, utilizing multiple pleiotropy testing methods, and applying various sensitivity analyses, we cannot entirely eliminate the potential impact of confounding variables on our results. Future advancements in algorithmic techniques may help further substantiate our findings. In addition, MDD is a clinically heterogeneous syndrome, and GWAS‐based case definitions may not capture important differences in symptom dimensions, illness course, severity, or biological subtypes.

## 5 Conclusion

In conclusion, this study supports potential causal associations between specific metabolites and MDD risk, with distinct immune cell subsets partially mediating these effects. These immune–metabolic pathways may help prioritize candidate biological pathways for future mechanistic and translational investigation, but confirmation in independent cohorts and mechanistic studies is still needed before clinical translation.

## Author Contributions

**Zhiwei Xu**: Conceptualization, Data curation, Formal analysis, Software, Visualization, Writing – original draft; **Yining Zhou**: Writing – review & editing, Software, Formal analysis, Visualization; **Xuecheng Zhang**: Conceptualization, Project administration, Writing – review & editing; **Xiaowen Chen**: Writing – review & editing; **Yajing Li, Jintao Wang, Ting Chang and Zhen Yuan**: Writing – original draft, Writing – review & editing; **Hongling Jia**: Conceptualization, Project administration, Supervision, Writing – review & editing.

## Funding

This work was supported by grant from National High‐level of Hospital Clinical Research Funding of China‐Japan Friendship Hospital (No. 2023‐NHLHCRF‐BQ‐46).

## Disclosure

Permission to reproduce material from other sources.

## Ethics Statement

The data for this study were derived from publicly available datasets, which were collected in compliance with local laws and ethical standards, including ethical approval and informed consent. Accordingly, no additional ethical approval is required for the data reanalysis presented in this study.

## Conflicts of Interest

The authors declare no conflicts of interest.

## Supporting information




**Supplementary Material**: brb371421‐sup‐0001‐FigureS1‐S6.docx

**Supplementary Material**: brb371421‐sup‐0002‐SuppMat.xlsx

## Data Availability

GWAS data for metabolites and immune cells can be accessed from the GWAS Catalog web (https://www.ebi.ac.uk/gwas/downloads/summary‐statistics). GWAS data for MDD were sourced from the Psychiatric Genomics Consortium (https://pgc.unc.edu/). The codes supporting the conclusions of this article will be made available by the corresponding author.
