# Supplementary material for: Elucidating Immune Cell Mediated Causal Pathways Linking Blood Metabolites to Major Depressive Disorder: A Mediation Mendelian Randomization Analysis
Source: Brain Behav. 2026 Jun 8;16(6):e71421. doi: 10.1002/brb3.71421 (PMC13247125; doi:10.1002/brb3.71421)
Supplement: Supplementary file 1 — Supplementary Material: brb371421‐sup‐0001‐FigureS1‐S6.docx [file BRB3-16-e71421-s002.docx]

Supplementary Material

## Supplementary Figures


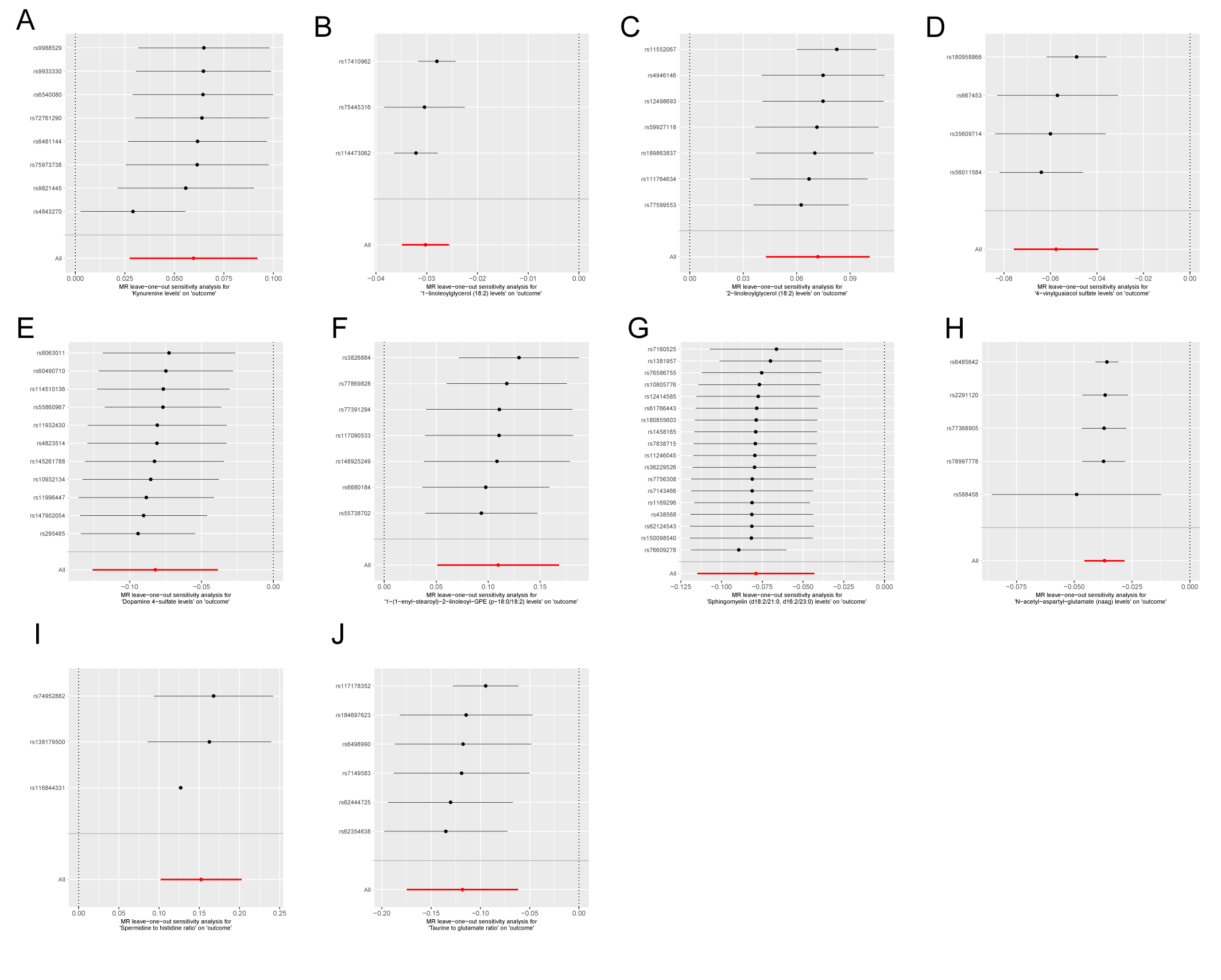


**Figure S1 Leave-one-out Plot**. (A) Kynurenine levels to MDD, (B) 1-linoleoylglycerol levels to MDD, (C) 2-linoleoylglycerol levels to MDD, (D) 4-vinylguaiacol sulfate levels to MDD, (E) Dopamine 4-sulfate levels to MDD, (F) 1-(1-enyl-stearoyl)-2-linoleoyl-GPE levels to MDD, (G) Sphingomyelin levels to MDD, (H) N-acetyl-aspartyl-glutamate levels to MDD, (I) Spermidine to histidine ratio to MDD, (J) Taurine to glutamate ratio to MDD.


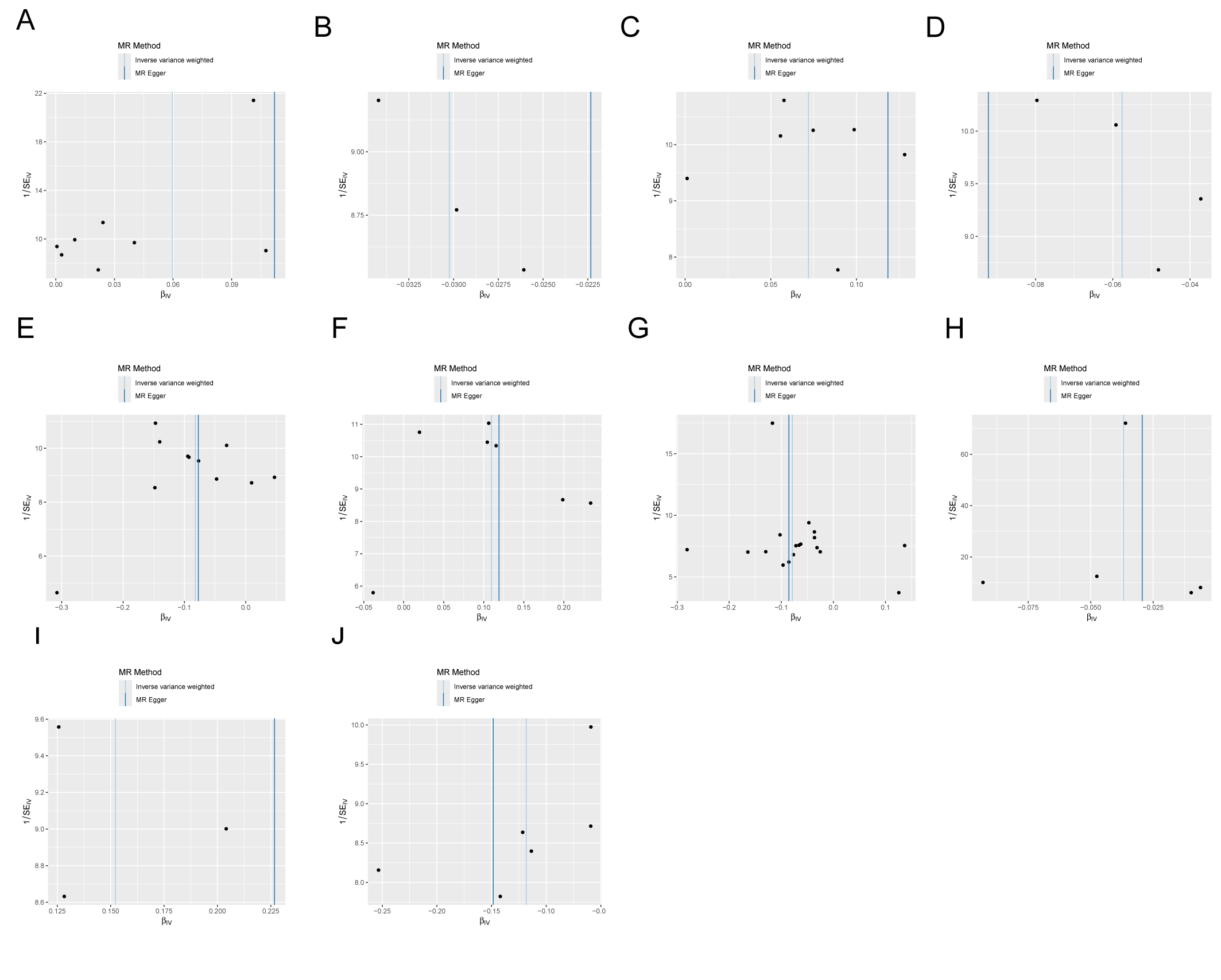


**Figure S2 Funnel Plot**. (A) Kynurenine levels to MDD, (B) 1-linoleoylglycerol levels to MDD, (C) 2-linoleoylglycerol levels to MDD, (D) 4-vinylguaiacol sulfate levels to MDD, (E) Dopamine 4-sulfate levels to MDD, (F) 1-(1-enyl-stearoyl)-2-linoleoyl-GPE levels to MDD, (G) Sphingomyelin levels to MDD, (H) N-acetyl-aspartyl-glutamate levels to MDD, (I) Spermidine to histidine ratio to MDD, (J) Taurine to glutamate ratio to MDD.


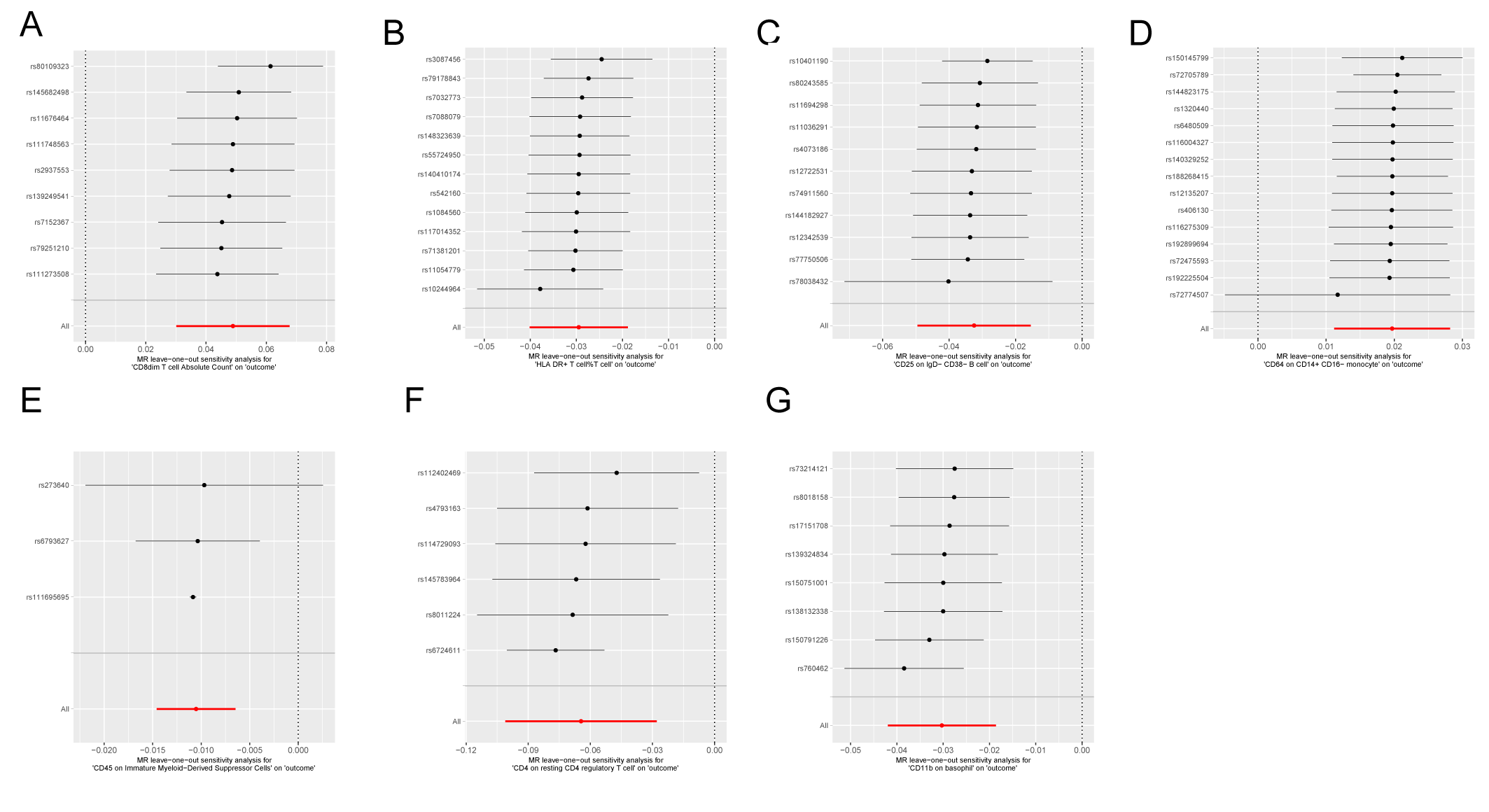


**Figure S3 Leave-one-out Plot**. (A) CD8dim T cell Absolute Count to MDD, (B) HLA DR+ T cell%T cell to MDD, (C) CD25 on IgD- CD38- B cell to MDD, (D) CD64 on CD14+ CD16- monocyte to MDD, (E) CD45 on Immature Myeloid-Derived Suppressor Cells to MDD, (F) CD4 on resting CD4 regulatory T cell to MDD, (G) CD11b on basophil to MDD.


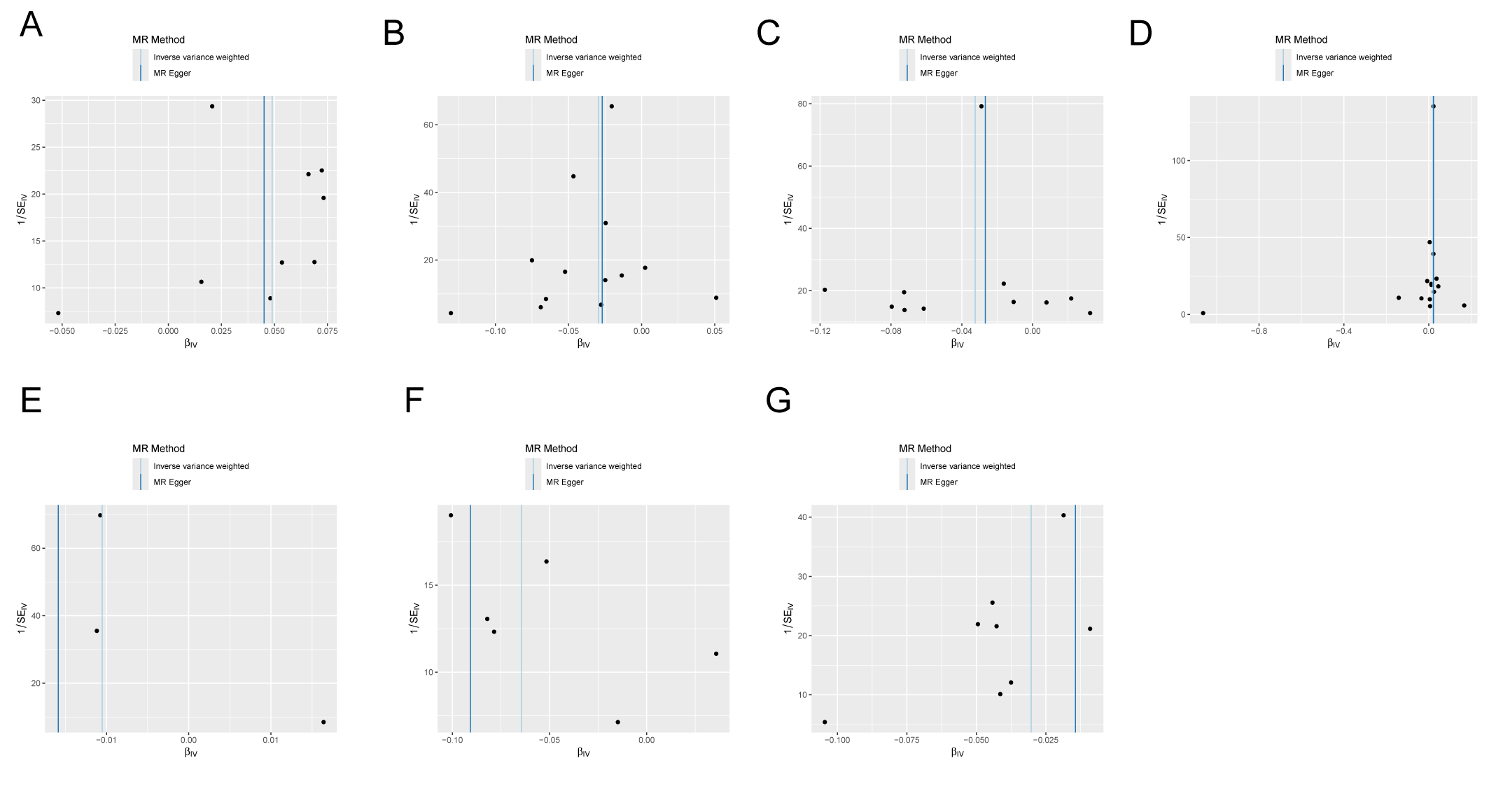


**Figure S4 Funnel Plot**. (A) CD8dim T cell Absolute Count to MDD, (B) HLA DR+ T cell%T cell to MDD, (C) CD25 on IgD- CD38- B cell to MDD, (D) CD64 on CD14+ CD16- monocyte to MDD, (E) CD45 on Immature Myeloid-Derived Suppressor Cells to MDD, (F) CD4 on resting CD4 regulatory T cell to MDD, (G) CD11b on basophil to MDD.


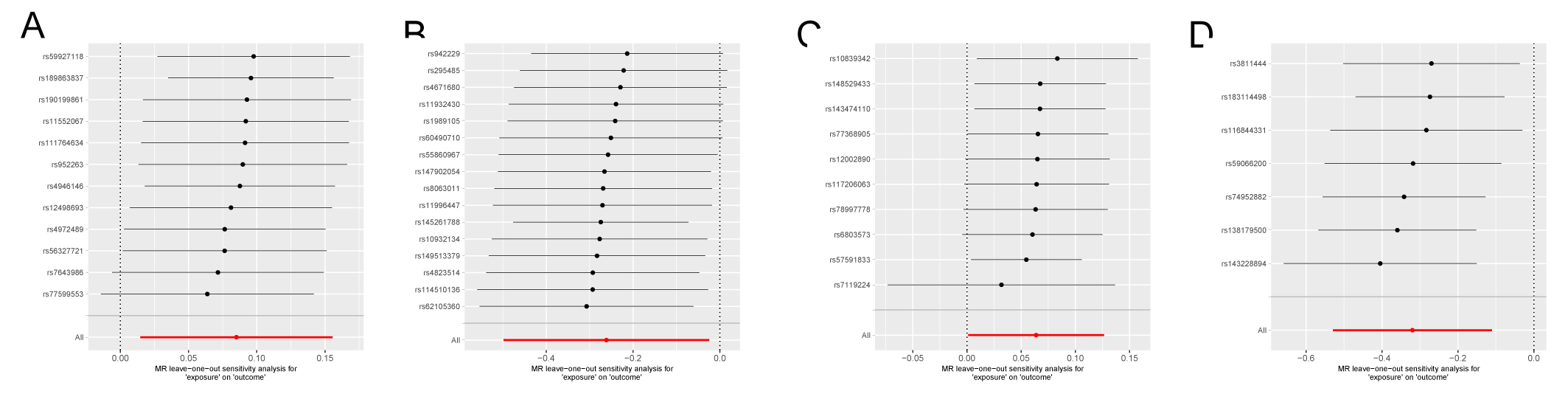


**Figure S5 Leave-one-out Plot**. (A) 2-linoleoylglycerol levels to CD8dim T cell Absolute Count, (B) Dopamine 4-sulfate levels to CD11b on basophil, (C) N-acetyl-aspartyl-glutamate levels to CD4 on resting CD4 regulatory T cell, (D) Spermidine to histidine ratio to CD4 on resting CD4 regulatory T cell.


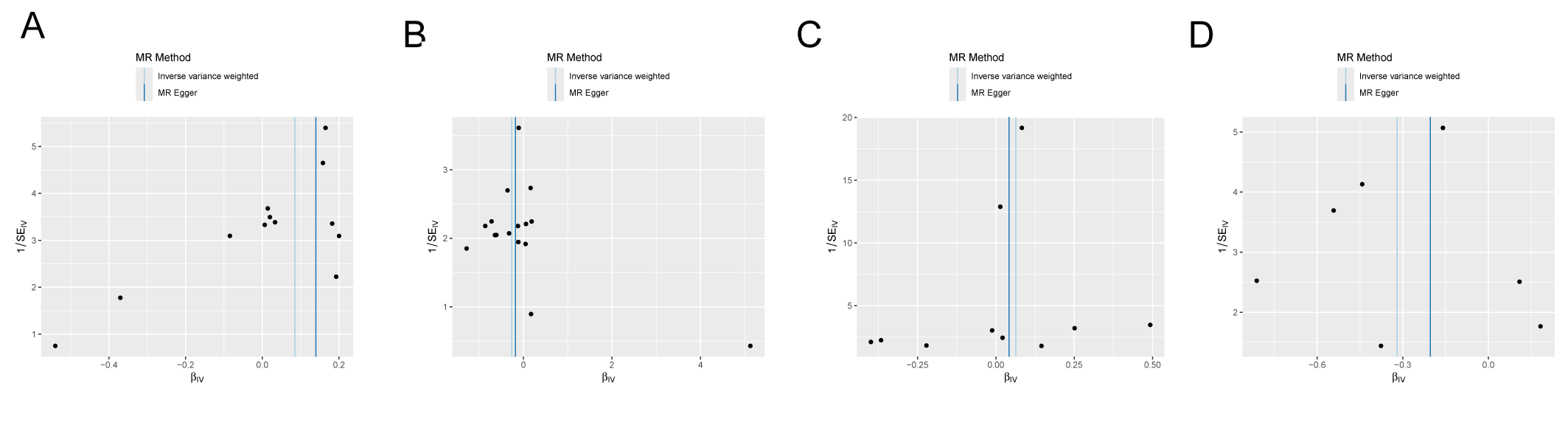


**Figure S6 Funnel Plot**. (A) 2-linoleoylglycerol levels to CD8dim T cell Absolute Count, (B) Dopamine 4-sulfate levels to CD11b on basophil, (C) N-acetyl-aspartyl-glutamate levels to CD4 on resting CD4 regulatory T cell, (D) Spermidine to histidine ratio to CD4 on resting CD4 regulatory T cell.
